# Venovenous extracorporeal membrane oxygenation devices-related colonisations and infections

**DOI:** 10.1186/s13613-017-0335-9

**Published:** 2017-11-07

**Authors:** Guillemette Thomas, Sami Hraiech, Nadim Cassir, Samuel Lehingue, Romain Rambaud, Sandrine Wiramus, Christophe Guervilly, Fanny Klasen, Mélanie Adda, Stéphanie Dizier, Antoine Roch, Laurent Papazian, Jean-Marie Forel

**Affiliations:** 1 Hôpital Nord, Réanimation des Détresses Respiratoires et des Infections Sévères, Assistance Publique–Hôpitaux de Marseille, 13015 Marseille, France; 2 URMITE, UMR CNRS 7278, Faculté de Médecine, Aix-Marseille Université, 13005 Marseille, France; 3 Hôpital de la Conception, Réanimation des brulés Assistance Publique–Hôpitaux de Marseille, 13005 Marseille, France; 4 Hôpital Nord, Service des Urgences, Assistance Publique–Hôpitaux de Marseille, 13015 Marseille, France

**Keywords:** Venovenous extracorporeal membrane oxygenation, Device-related infections, Device-related colonisation, Infection rate, Colonisation rate

## Abstract

**Background:**

Nosocomial infections occurring during extracorporeal membrane oxygenation (ECMO) support have already been reported, but few studied infections directly related to ECMO devices. This study aims to evaluate the rate of both colonisations and infections related to ECMO devices at the time of ECMO removal.

**Results:**

We included all consecutive adult patients treated with venovenous ECMO (VV-ECMO) for at least 48 h during a 34-month study. At the time of ECMO removal, blood cultures, swab cultures on insertion cannula site and intravascular cannula extremity cultures were systematically performed. Each ECMO device was classified according to the infectious status into three groups: (1) uninfected/uncolonised ECMO device, (2) ECMO device colonisation and (3) ECMO device infection. Ninety-nine patients underwent 103 VV-ECMO, representing 1472 ECMO days. The ECMO device infection rate was 9.7% (10 events), including 7 ECMO device-related bloodstream infections (6.8%). The ECMO device colonisation rate was 32% (33 events). No difference was observed between the three groups, regarding days of mechanical ventilation, ICU length of stay, ICU mortality and in-hospital mortality. We observed a longer ECMO duration in the ECMO device colonisation group as compared to the uninfected/uncolonised ECMO device group [12 (9–20 days) vs. 5 days (5–16 days), respectively, *p* < 0.05].

**Conclusions:**

At the time of ECMO removal, systematic blood culture and intravascular extremity cannula culture may help to diagnose ECMO device-related infection. We reported a quite low infection rate related to ECMO device. Further studies are needed to evaluate the benefits of systematic strategies of cannula culture at the time of ECMO removal.

**Electronic supplementary material:**

The online version of this article (10.1186/s13613-017-0335-9) contains supplementary material, which is available to authorized users.

## Background

Venovenous extracorporeal membrane oxygenation (VV-ECMO) has become a widely accepted treatment option for life-threatening acute respiratory failure when mechanical ventilation (MV) and adjunctive measures fail to provide adequate gas exchange or when lung rest cannot be achieved due to high ventilator requirements [1, 2]. Over the last two decades, the technique has improved significantly, and several studies have reported encouraging survival rates using VV-ECMO in adults with acute respiratory distress syndrome (ARDS) [3–6]. However, major adverse events have been described, among which infections seem to be the most frequent [1, 6]. In 2011, the Extracorporeal Life Support Organization (ELSO) reported an incidence of 11.7% proven infections in 20,741 ECMO cases for a rate of 15.4 per 1000 ECMO days [7]. To date, most of the studies described nosocomial infections or bloodstream infections (BSI) occurring during ECMO support, but very few studied infections directly related to ECMO devices. Moreover, these studies often mixed venovenous and venoarterial ECMO support, which are very different devices regarding the type of patients, the duration of ECMO and the cannulation procedure [8, 9].

The main objective of this study was to evaluate the rates of both infections and colonisations related to ECMO devices in VV-ECMO adult patients at the time of ECMO removal.

## Methods

### Study design

An epidemiologic, prospective, observational study was conducted in the 14-bed medical intensive care unit (ICU) of a teaching hospital (Hôpital Nord, Marseille, France), a regional referral centre for the treatment of acute severe respiratory failure. The study was approved by the ethical committee of the «Société de Réanimation de Langue Française». According to French law, no consent for the study was required because it did not modify existing diagnostic or therapeutic strategies.

### Patients and ECMO indications

We prospectively included all consecutive adult patients treated with VV-ECMO for at least 48 h during a 34-month study. The ECMO-based programme includes a mobile unit that is able to initiate ECMO in referring hospitals of our region (Provence-Alpes-Côte d’Azur) before transfer to our ECMO referral centre [5].

The decision to initiate ECMO is based on the following: persistent hypoxaemia, defined as PaO_2_/FiO_2_ ≤ 70 mmHg for at least 6 h under FiO2 at 1 despite optimisation of mechanical ventilation or PaO_2_/FiO_2_ < 100 mmHg with a Pplat value greater than 35 cmH_2_O or respiratory acidosis with pH ≤ 7.15 despite a respiratory rate greater than 35/min. Exclusion criteria for ECMO included the following: any contraindications to heparin treatment, Sequential Organ Failure Assessment (SOFA) score > 16 [10], moribund patients or those with decisions to limit therapeutic interventions.

### ECMO protocol

Venovenous ECMO was instituted using percutaneous cannulation by cardiac surgeons, typically in a femoral–jugular configuration but also in femoral–femoral configuration, especially when ECMO was used as a bridge to lung transplantation. We used centrifugal pumps (Bio-console 560; Medtronic Perfusion Systems, Minneapolis, MN, USA) with a flow of 3–5 L/min in all patients. Circuits were heparin-coated and composed of Quadrox D with Bioline Coating oxygenators (Maquet, Hirrlingen, Germany), 17–25-Fr cannulae (Edwards Lifesciences, Irvine, CA, USA) and intersept polyvinyl chloride (PVC) class VI tubing (Medtronic, Watford, Hertfordshire, UK).

All cannulas were inserted using strict sterile precautions consistent with Healthcare Infection Control Practices Advisory Committee guidelines (HICPAC guidelines) [11]. For each patient, the cannula insertion site was cleansed with 96% ethanol solution containing 5% povidone-iodine. Sterile drapes were placed over the insertion site. No specific antibioprophylaxis was used at the time of cannulation. Occlusive dressings were used.

A highly trained ICU nursing staff achieved standardised cannula care every 72 h or earlier if clinically indicated (dirty or bloody dressing). If necessary (haemolysis, fibrinolysis), the ECMO circuit was changed using strict sterile precautions as detailed above.

When the ECMO was removed, specimens were systematically collected as follows: (a) blood cultures were sampled from the central venous catheter (CVC), arterial catheter and post-membrane oxygenator. The blood culture vials used for aerobic and anaerobic cultures (Bactec; Becton–Dickinson, Sparks, MD, USA) were incubated for 5 days. After the incubation period and automatic culture detection (Bactec 9240; Becton–Dickinson, Sparks, MD, USA), Gram staining was performed, and the samples were cultured on 5% sheep blood and chocolate agar plates at 37 °C under aerobic and anaerobic atmospheric conditions for all positive blood cultures. (b) Swabs were sampled on the drainage and return cannula site skin just before cleaning with 5% povidone-iodine antiseptic. The culture technique is described as follows. (c) The intravascular extremity of the drainage and return cannula were cut in a sterile manner and analysed by a culture technique described as follows. The extremities of the cannulas, the central venous and the arterial catheter tips when removed, were mixed with tryptic soy broth; 10 μL of each mixture was then cultured on chocolate agar plates at 37 °C under aerobic atmospheric conditions. Swab samples were semi-quantitatively processed immediately by streaking the entire surface of the plates. Identification was performed when a culture yielded at least 10^3^ colony-forming units (CFU)/mL. Matrix-assisted laser desorption/ionisation time-of-flight mass spectrometry (MALDI-TOF MS) was used for the bacterial identification as previously described [12].

### Definition of ECMO device colonisation and infection

The definitions of ECMO device colonisation or infection were adapted from French and American central line-associated bloodstream infection guidelines [13, 14]. These definitions concern the central line defined as an intravascular catheter that terminates at or close to the heart or in one of the great vessels excluding ECMO devices. Thus, the following definitions were used: (a) cannula colonisation (CC) was defined as a positive quantitative intravascular extremity culture (≥ 10^3^ CFU). (b) Skin colonisation (SC) was a positive quantitative swab culture (≥ 10^3^ CFU). (c) Not related ECMO device bacteraemia was defined as one or more positive blood cultures with negative cannula colonisation and another infectious site responsible for bacteraemia. (d) Contamination was defined as one positive blood culture for common skin contaminants [14, 15]. (e) ECMO device infection (ED-I) corresponded to: (e.1) ECMO device-related blood stream infection (EDR-BSI), which was a combination of one or more positive blood cultures (from the CVC, arterial catheter or post-membrane oxygenator) sampled immediately before or within 48 h after ECMO removal, a quantitative intravascular cannula extremity positive culture for the same micro-organism(s) and no other infection explaining the positive blood culture; (e.2) cannula infection (CI), which was considered in cases of a positive quantitative intravascular cannula extremity culture, negative blood culture and systemic infectious signs in the absence of any other infection. CI was also considered in case of a positive quantitative intravascular cannula extremity culture and local infection signs (local purulence or infection signs at insertion site); (e.3) in patients with blood culture and/or quantitative intravascular cannula portion culture positive for coagulase-negative staphylococci, EDR-BSI and CI were considered depending on clinical features (fever, sepsis, septic shock) within 48 h after ECMO removal and on clinical evolution under specific treatment if introduced by the clinician.

An adjudication committee, including one infectious disease specialist and three intensivists, retrospectively classified each case into three categories: (1) uninfected/uncolonised ECMO device (U-I/C ED), including sterile samples, skin colonisation, blood culture contamination and not related ECMO device bacteraemia; (2) ECMO device colonisation (ED-C), including cannula colonisation associated or not with skin colonisation; (3) ECMO device infection (ED-I), including EDR-BSI and CI as previously defined.

### Collected data

Prospectively collected data included demographic data; body mass index (BMI); severity of illness as assessed by the Simplified Acute Physiology Score (SAPS) II [16] and SOFA score at ICU admission [10]; major comorbidities; indication for ECMO; site of cannulation; site of ECMO implantation; ECMO system exchange; ECMO transfusion (blood, platelets and plasma), pre- and per-ECMO steroid use; pre- and per-ECMO antibiotics use; duration of both ECMO and mechanical ventilation; outcome (ICU and hospital mortalities, ventilator and ECMO-free days at both day 28 and day 90, ICU length of stay); and nosocomial infections, primary bloodstream infections or fungaemia during ECMO support. Nosocomial infection definitions agreed with those of the Centers for Disease Control and Prevention National Nosocomial Infections Surveillance System [17]. Ventilator-associated pneumonia (VAP) was diagnosed according to previously published criteria [18].

### End points

The primary end points were the rates of ECMO device infection or colonisation at the time of ECMO removal. Secondary end points were the rate of skin colonisation and outcomes, such as ICU length of stay, ICU mortality, in-hospital mortality and day-90 mortality, day-28 and day-90 ventilator and ECMO-free days.

### Statistical analysis

Descriptive statistics included percentages for categorical variables and medians and interquartile ranges for continuous variables. Comparisons between the three categories (U-I/C ED, ED-C and ED-I) for continuous variables were made using the Kruskal–Wallis test with a post hoc method for multiple comparisons (step-up Simes method to calculate adjusted *p* value). Comparisons between the three categories (U-I/C ED, ED-C and ED-I) for categorical variables were made using the Pearson Chi-square test for trend. A multinomial logistic regression procedure was performed to identify factors associated with ED-I or ED-C. The U-I/C ED group was used as the reference group. All of the variables with *p* value < 0.20 (gender, body mass index, statin therapy, per-ECMO plasma transfusion, reason for ECMO, location of ECMO cannulation, type of cannulation, pre-ECMO antibiotic and ECMO duration) were included in the model. The Fleiss’ kappa was calculated to evaluate the reliability agreement between the 4 experts regarding the classification of each ECMO case. A *p* value < 0.05 was considered significant. The statistical analysis was conducted using SPSS, version 20.0 (NY, USA).

## Results

### Patients

**Fig. 1:**
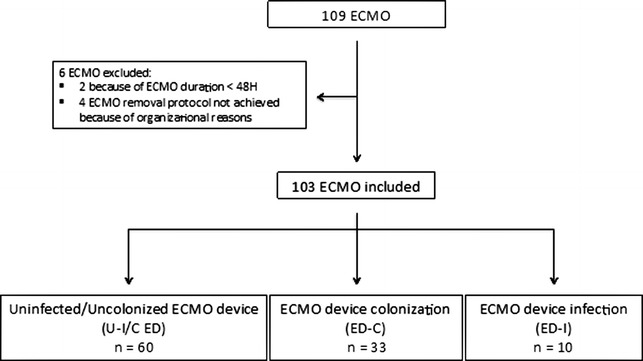
Study flow chart. ECMO, extracorporeal membrane oxygenation

During the study period, 105 patients underwent 109 VV-ECMO (Fig. 1). Four patients underwent 2 VV-ECMO during the same ICU stay with an interval of at least 2 days between each ECMO implantation. Finally, 103 VV-ECMO were analysed (representing 1472 ECMO days). The median age was 49 years (38–62), and the most frequent reason for ECMO was ARDS (77.6%). Fifty-three patients were referred to our ECMO centre and transported by our mobile team. The median ECMO duration was 11 days (6–18 days). A total of 63 circuit changes were done in 38 VV-ECMO. General characteristics of each group are provided in Table 1.

**Table 1:** General characteristics of ECMO in patients without infected/colonised ECMO device, with ECMO device colonisation and ECMO device infection (at the time of ECMO removal)

Number of ECMO^a^	U-I/C ED	ED-C	ED-I	*P* value^b^
	60	33	10	
Age (years)	48 (37–61)	57 (47–63)	43 (41–63)	0.278
Male (*n*, %)	35 (58.3)	23 (69.7)	8 (80)	0.122
BMI (kg/m^2^)	24 (22–29)	27 (24–31)	26 (24–27)	0.183
SOFA score^c^	10 (7–12)	9 (7–14)	9 (7–10)	0.607
SAPS II^c^	44 (39–56)	47 (38–57)	46 (36–53)	0.833
Underlying condition				
Diabetes mellitus	9 (15)	4 (12.1)	0	0.233
Renal insufficiency	0	2 (6.1)	0	0.300
Immunocompromised^d^	5 (8.3)	6 (18.2)	1 (10)	0.402
COPD	5 (8.3)	2 (6.1)	0	0.349
Solid tumour	9 (15)	4 (12.1)	2 (20)	0.906
Cirrhosis	2 (3.3)	0	0	0.272
Statin therapy	8 (13.3)	2 (6.1)	0	0.118
ICU stay before ECMO centre admission	2 (0–8)	5 (1–10)	3 (0–4)	0.237
Reason for ECMO^e^				
ARDS	42 (70)	28 (84.8)	10 (100)	0.011*
CAP	18	13	5 (50)	
NP	20	13	4 (40)	
Extrapulmonary	4	2	1 (10)	
Bridge to lung transplantation	2 (6.7)	2 (6.1)	0	0.965
Primary graft dysfunction	14 (23.3)	3 (9.1)	0	0.023*
ECMO characteristics				
Mobile ECMO team	27 (45)	21 (63.6)	5 (50)	0.272
Location of ECMO cannulation				
ICU	44 (73.3)	31 (93.9)	10 (100)	0.005^#^*
Operating room	16 (26.7)	2 (6.1)	0	
Per-ECMO blood transfusion	9 (4–21)	8 (5–17)	9 (6–16)	0.934
Per-ECMO platelet transfusion	1 (0–4)	1 (0–4)	1 (0–1)	0.651
Per-ECMO plasma transfusion	2 (0–10)	0 (0–4)	0 (0–4)	0.197
Pre-ECMO steroids	14 (23.3)	8 (24.2)	2 (20)	0.903
Per-ECMO steroids	31 (51.7)	15 (45.5)	3 (30)	0.214
Pre-ECMO antibiotics^f^	48 (80)	29 (87.9)	10 (100)	0.085
Per-ECMO antibiotics^g^	58 (96.7)	33 (100)	10 (100)	0.272
Antibiotics at the time of ECMO removal	48 (80)	30 (90.9)	5 (50)	0.314
BSI during ECMO^h^	10 (16.7)	8 (24.2)	2 (20)	0.525
Cannulation				
Femoro–femoral	19 (31.7)	5 (15.2)	0	0.011*
Femoro-jugular	41 (68.3)	28 (84.8)	10 (100)	
ECMO circuit change (≥ 1)	24 (40)	12 (36.4)	2 (20)	0.278
ECMO duration (days)	7.5 (5–16)	12 (9–20)	13 (11–17)	0.021^#^

Data are provided as no. (%) of ECMO or median value (interquartile range)

*ARDS* acute respiratory distress syndrome, *BMI* body mass index, *CAP* community-acquired pneumonia, *COPD* chronic obstructive pulmonary disease, *ECMO* extracorporeal membrane oxygenation, *ICU* intensive care unit, *NP* nosocomial pneumonia, *SAPS II* Simplified Acute Physiology Score, *SOFA* sepsis-related organ failure assessment, *U-I/C ED* uninfected/uncolonised ECMO device, *ED-C* ECMO device colonisation, *ED-I* ECMO device infection

* *p* < 0.05, comparison between U-I/C ED and ED-I

^#^ *p* < 0.05, comparison between U-I/C ED and ED-C

^a^Among the 99 patients, 4 underwent 2 ECMO during their ICU stay corresponding to 103 VV-ECMO

^b^ *p* value corresponds to the comparison between the three categories (U-I/C ED, ED-C, ED-I)

^c^Calculated at ICU admission

^d^Includes patients with human immunodeficiency virus, solid organ transplantation or haematological malignancy and those receiving chemotherapy, immunosuppressive agents or long-term corticosteroid therapy

^e^For 2 patients, ECMO reason was thoracic surgery

^f^Pre-ECMO antibiotics correspond to antibiotics received for at least 24 h before ECMO implantation

^g^Per-ECMO antibiotics correspond to antibiotics received immediately after ECMO implantation

^h^Bloodstream infection (BSI) under ECMO includes primary and secondary bloodstream infections

### ECMO device colonisation and infection (Fig. 2)

At the time of ECMO removal, the rate of ECMO device colonisation (ED-C) was 32% (33 events). The rate of ECMO device infection (ED-I) was 9.7% (10 events), including 7 ECMO device-related bloodstream infections (6.8%). No patient presented with insertion site infectious signs at any moment during ECMO support or within the 48-h period following ECMO removal. The uninfected/uncolonised ECMO device (U-I/C ED) rate was 58.3% (60 of 103). Fleiss’ kappa coefficient was 0.94 (standard error, 0.04), corresponding to strong agreement between the experts regarding the classification.

**Fig. 2:**
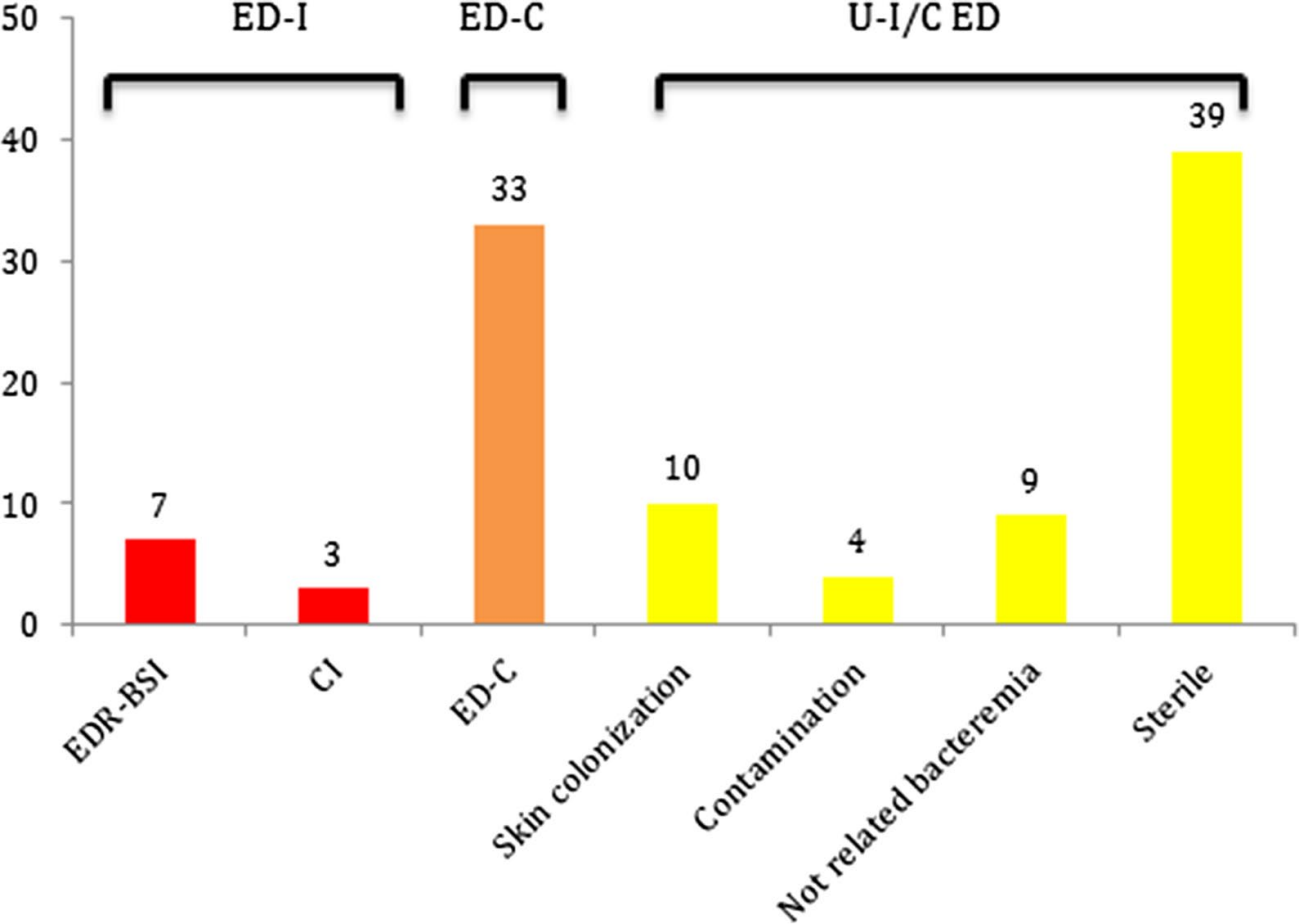
Details of each ECMO samples leading to different categories: ECMO device infection (ED-I) including ECMO device-related bloodstream infections (EDR-BSI) and cannula infections (CI); ECMO device colonisation (ED-C); uninfected/uncolonised ECMO device (U-I/C ED) including skin colonisation, contamination, not related bacteremia and sterile samples

**Fig. 3:**
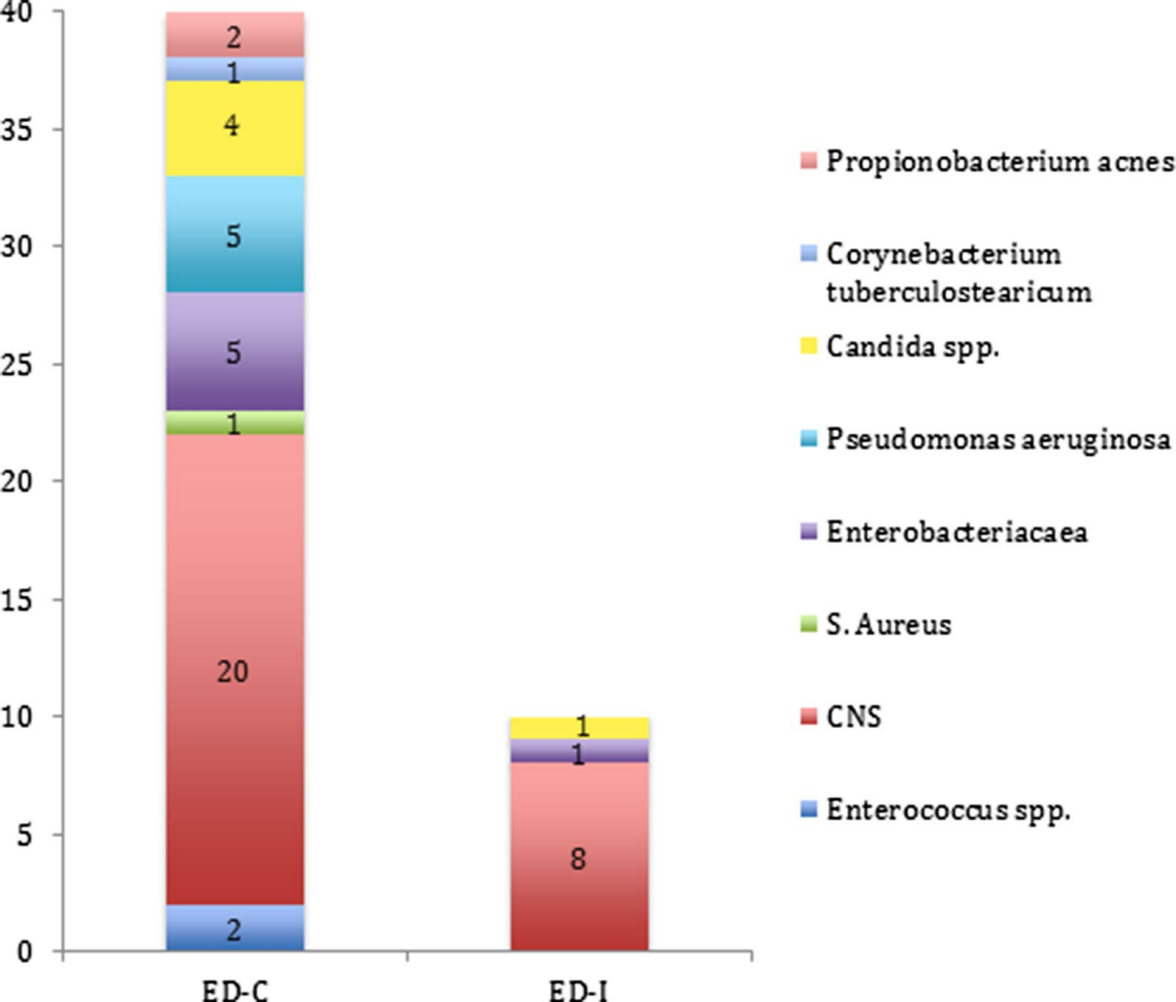
Micro-organisms associated with ECMO device colonisations and infections. *CNS* Coagulase-negative staphylococci. Seven cannulas were colonised with two different micro-organisms corresponding to 33 cannula colonisations and a total number of 40 micro-organisms

A total of 127 femoral cannulas were inserted. In all, 22% (28 of 127) of the cultures were positive for at least one micro-organism compared with 35.4% of the cultures (28 of 79) regarding the jugular site (*p* = 0.052). Micro-organisms responsible for ED-C and ED-I are detailed in Fig. 3 and Additional file 1: Table S1. Coagulase-negative staphylococcus was the most frequent organism responsible for ED-I (8/10, 80%) and ED-C (20/33, 60.6%). The details of blood culture results are summarised in Additional file 2: Table S2.

We observed a longer ECMO duration in the ED-C group compared with the U-I/C ED group [12 days (9–20 days) versus 5 days (5–16 days), respectively, *p* < 0.05]. Using multivariate analysis, we did not identify any factor associated with ED-C or ED-I (Additional file 3: Table S3).

### Skin colonisation

The skin colonisation rate was 23.3% (24 events). When cannula colonisation was observed, concomitant skin colonisation was present in 42.4% of cases (14 of 33), with the same micro-organism in 71.4% of cases (10 of 14). Considering ED-I, no concomitant skin colonisation was observed. No differences were noted regarding skin colonisation between femoral and jugular sites (22/127, 17.3%; 14/79, 17.7%, respectively, *p* = 0.91).

### Outcome

Fifty-two patients died in the ICU (50.5%) and 58 during hospitalisation (56.3%). No difference was observed among the three categories (U-I/C ED, ED-C and ED-I) regarding ICU length of stay, ICU mortality, in-hospital mortality and day-90 mortality (Table 2).

**Table 2:** Outcome of extracorporeal membrane oxygenation in patients without infected/colonised ECMO device, with ECMO device colonisation and ECMO device infection (at the time of ECMO removal)

Number of ECMO^a^	U-I/C ED	ED-C	ED-I	*p* value
	60	33	10	
ICU LOS	25 (14–39)	24 (20–33)	30 (23–37)	0.614
ICU mortality	27 (45)	21 (63.6)	4 (40)	0.559
Day-90 mortality	28 (46.7)	22 (66.7)	4 (40)	0.622
In-hospital mortality	32 (53.3)	22 (66.7)	6 (60)	0.896
Days of MV	23 (12–37)	24 (18–32)	28 (23–30)	0.671
VFD day 28	0 (0–1)	0 (0–0)	0 (0–0)	0.385
VFD day 90	1 (0–60)	0 (0–35)	9 (0–52)	0.506
ECMOFD day 28	13 (0–21)	3 (0–18)	7 (0–14)	0.169
ECMOFD day 90	63 (0–83)	9 (0–78)	62 (0–76)	0.156
ECMO weaning	43 (71.7)	17 (51.5)	7 (70)	0.283

Data are provided as no. (%) of ECMO or median value (interquartile range)

*ECMO* extracorporeal membrane oxygenation, *ECMOFD* ECMO-free day, *ICU* intensive care unit, *LOS* length of stay, *MV* mechanical ventilation, *VFD* ventilator-free day, *U-I/C ED* uninfected/uncolonised ECMO device, *ED-C* ECMO device colonisation, *ED-I* ECMO device infection

^a^Among the 99 patients, 4 underwent 2 ECMO during their ICU stay corresponding to 103 VV-ECMO

A total of 27 patients underwent at least one nosocomial infection other than ED-I, without differences among the three groups (*p* = 0.30). Forty-four nosocomial infections, excluding ED-I, occurred during the 103 VV-ECMO supports, corresponding to 29.9 infectious episodes per 1000 ECMO days. Ventilator-associated pneumonia (VAP) was the most frequent nosocomial infection (45.5%), followed by primary bloodstream infection (36.4%) (Additional file 4: Table S4).

## Discussion

To our knowledge, this is the first study that systematically analysed ECMO devices at the time of ECMO removal, providing the rate of ECMO device-related infection and colonisation in an adult cohort of venovenous ECMO supports. Our results indicate that the ECMO device infection rate (cannula infections or ECMO device-related bloodstream infections) was 9.7%, and the ECMO device colonisation rate was 32%. Indeed, most of the studies related the incidence of nosocomial infections and bloodstream infections while on venoarterial or venovenous ECMO support, and these studies often used paediatric cohorts [7–9, 19, 20]. The originality of our work was to assess the infection and colonisation rates directly associated with the ECMO device. Schmidt et al. [9] reported a large series of 220 venoarterial ECMO support cases in adult cardiogenic shock and described 21 (9.5%) cannula infections, defined as the association of local signs of infection at the access site with a positive culture of subcutaneous needle aspirate from the cannula site. One major difference with this study is that VA-ECMO cannulation was performed with an invasive surgical procedure, especially in the case of central VA-ECMO, whereas VV-ECMO cannulation only requires a percutaneous procedure (which was the case for all of our patients). Furthermore, the definition of cannula infection was different and could reflect surgical site infection rather than cannula infection in this study. Of note, none of our patients presented with cannula local infectious signs at any moment during ECMO support and even during the 48 following hours. Lubnow et al. described technical complications leading to system exchange in 265 adult patients treated with VV-ECMO support for acute respiratory failure. Eighty-three patients underwent at least one system change, and 4 of these cases (5%) were due to suspected infection [21]. More recently, Hahne et al. evaluated the culture results of 186 cannulae removed from 94 patients who benefited from extracorporeal circulation for lung or cardiac assistance. Fifteen patients (16%) presented cannula-related infection [22].

ECMO device-related infections may involve the drainage cannula, the return cannula or the membrane oxygenator (MO). Thus, Kuehn et al. [23] hypothesised that the artificial surfaces of the ECMO circuit, particularly the MO, could be the target of microbial adhesion and colonisation, favouring the development of ECMO-related bloodstream infection. The overall patient-based positivity by PCR was 45%. In the present study, membrane oxygenator infection was difficult to assess. We performed post-membranous blood culture on the day of removal (Additional file 4: Table S4), which was positive in 15 cases and allowed the diagnosis of ECMO device-related bloodstream infection (EDR-BSI) in only one case.

Our results revealed a longer ECMO duration in the ED-C group compared with the U-I/C ED group. Catheter duration is a well-known risk factor for catheter colonisation or catheter-related bloodstream infection [24]. Moreover, the prevalence of nosocomial infection increases with ECMO duration [7, 9]. No difference was observed between the U-I/C ED group and ED-I, which is probably due to the small number of patients in this group. At least, skin colonisation was not observed in the 10 ED-I, suggesting that ECMO device-related infections could originate from haematogenous contamination or circuit changes although our data cannot confirm this hypothesis (Table 1).

We reported a higher proportion of primary graft dysfunction, femoro–femoral cannulation and cannulation in the operating room in the U-I/C ED group compared with the ED-C group and/or the ED-I group. This finding can be easily explained by the fact that all patients with primary graft dysfunction benefited from ECMO cannulation at the end of the lung transplant, in the operating room, and with femoro–femoral cannulation due to surgical technical reasons. Moreover, the duration of ECMO was reduced for primary graft dysfunction indication compared with ARDS indication [5 (4–6) vs. 12 days (7–18), *p* < 0.001 data not shown]. Thus, the differences observed between the different groups are probably the result of significant differences of ECMO duration.

Our study described a very much higher rate of infection with ECMO device than with central venous catheter. During the study period, central venous catheter-related infection rate was 1.2 per 1000 catheter days. ECMO cannula size is bigger (17–25 Fr) and duration of ECMO longer than central venous catheter. Moreover, cannula change is highly problematic because of the few vascular accesses and due to patient’s vital dependence on ECMO support.

 Antibiotic prophylaxis to prevent nosocomial infections in ECMO patients remains highly controversial due to the emergence of resistance to antibiotics and *Clostridium difficile*-associated colitis [25]. Daily surveillance blood cultures have been proposed as an alternative to antibiotic prophylaxis and remain a routine practice in many ECMO centres, but this strategy is costly and resource consuming [9, 19, 26]. Wide variability in practice is also noted regarding the prevention of nosocomial and bloodstream infections for patients requiring ECMO. In a recent survey of the ELSO members interested in nosocomial bloodstream infection prevention practice, only one-quarter of respondents reported the use of a bundle or checklist during ECMO cannula insertion and less than half utilise a bundle or checklist for cannula maintenance [27]. In our opinion, if daily surveillance blood culture in patients with ECMO support should not be recommended, the systematic culture of vascular cannula portions associated with blood culture performed at the time of removal may help the clinician to make the diagnosis of ECMO device-related infection and guide the antibiotics prescription. Finally, we did not use chlorhexidine antiseptic protocol at the time of cannula insertion and for the standardised cannula care. Chlorhexidine-impregnated dressing for cannula dressing was not used in our study which could be evaluated in the future to evaluate the impact on the rate of ECMO device colonisation and infection.

 Our study presents several limitations. There is currently no consensus on the definition of ECMO device-related infection. Indeed, the Centers for Disease Control and Prevention (CDC) guidelines have established a clear definition of central line-related infection or blood stream infections that precisely exclude ECMO devices [14]. Moreover, the definitions that include differential time to blood culture positivity appear to be inappropriate given the impossibility and danger of performing blood culture in ECMO cannulae [13, 28]. We have decided to use the threshold of 10^3^ colony-forming unit for the positivity of the device culture, which is derived from catheter infection literature but not validated for the ECMO device so far. At least, our study focused on ECMO device infection and colonisation at the time of removal and not during the ECMO support period. Moreover, the huge majority of our patients were receiving antibiotics at the time of ECMO insertion and during the ECMO run. These two last elements could have led to possible underestimation of the number of infections and colonisations. We did not collected data regarding dressing disruption or changes that might help to explain the differences between the rate of colonisation and the rate of infection. Using a multinomial logistic regression procedure, we failed to establish factors associated with ECMO device colonisation or infection, probably because of the cohort size.

## Conclusions

 At the time of ECMO removal, systematic blood culture and intravascular extremity cannula culture may help to diagnose ECMO device-related infection. We reported a quite low infection rate related to the devices. Further studies are needed to evaluate the benefits of systematic strategies of cannula culture at the time of ECMO removal.

## Additional files

**Additional file 1.** **Table S1**. Microorganisms cultured in different samples of 103 Extracorporeal Membrane Oxygenation (at the time of ECMO removal).

**Additional file 2.** **Table S2**. Microorganisms cultured in blood culture (at the time of ECMO removal).

**Additional file 3.** **Table S3**. Nosocomial infections during ECMO support of Extracorporeal Membrane Oxygenation (ECMO) in patients without infected/colonized ECMO device (U-I/C ED), with EMCO device colonization (ED-C) and ECMO device infection (ED-I) (at the time of ECMO removal).

**Additional file 4.** **Table S4**. Multivariate analysis of factors associated with ECMO device infection (ED-I) or colonization (ED-C) at the time of ECMO removal.

## Abbreviations

ARDS: acute respiratory distress syndrome
BC: blood culture
BMI: body mass index
BSI: bloodstream infections
CC: cannula colonisation
CI: cannula infection
CFU: colony-forming units
CVC: central venous catheter
ELSO: Extracorporeal Life Support Organization
ECMO: extracorporeal membrane oxygenation
ED-C: ECMO device colonisation
ED-I: ECMO device infection
EDR-BSI: ECMO device-related blood stream infection
HICPAC Guidelines: Healthcare Infection Control Practices Advisory Committee Guidelines
ICU: intensive care unit
MALDI-TOF MS: matrix-assisted laser desorption/ionisation time-of-flight mass spectrometry
MV: mechanical ventilation
PEEP: positive end-expiratory pressure
Pplat: plateau pressure
SAPS: Simplified Acute Physiology Score
SK: akin colonisation
SOFA: Sequential Organ Failure Assessment
U-I/C ED: uninfected/uncolonised ECMO device
VAP: ventilator-associated pneumonia
VT: tidal volume
VV-ECMO: venovenous extracorporeal membrane oxygenation
