# Supplementary material for: Venovenous extracorporeal membrane oxygenation devices-related colonisations and infections
Source: Ann Intensive Care. 2017 Nov 7;7:111. doi: 10.1186/s13613-017-0335-9 (PMC5676570; doi:10.1186/s13613-017-0335-9)
Supplement: Supplementary file 1 — Additional file 1. Table S1. Microorganisms cultured in different samples of 103 Extracorporeal Membrane Oxygenation (at the time of ECMO removal). [file 13613_2017_335_MOESM1_ESM.doc]

**Table S1:** Microorganisms cultured in different samples of 103 Extracorporeal Membrane Oxygenation

|  | Drainage cannula  swab culture  n = 99 ^a^ | Return cannula swab culture  n = 103 | Blood culture ^b^  n = 309 | Drainage cannula culture  n = 103 | Return cannula culture  n = 103 |
| --- | --- | --- | --- | --- | --- |
| Gram positive |  |  |  |  |  |
| *CNS* | 2 | 6 | 11 | 18 | 22 |
| *Enterococcus spp* | 1 | 2 | 8 | 1 | 2 |
| *S. aureus* | 1 | 0 | 4 | 1 | 1 |
| *Bacillus spp* | 0 | 1 |  | 0 | 0 |
| *Propionobacterium acnes* | 0 | 0 | 1 | 2 | 2 |
| *Corynebacterium spp* | 0 | 0 | 1 | 1 | 0 |
|  |  |  |  |  |  |
| Gram negative |  |  |  |  |  |
| *Enterobacter spp.* | 5 | 2 | 4 | 2 | 2 |
| *Pseudomonas aeruginosa* | 4 | 3 | 5 | 3 | 2 |
| *Morganella morganii* | 1 | 1 | 0 | 1 | 0 |
| *Klebsiella spp* | 1 | 1 | 2 | 0 | 1 |
| *Escherichia coli* | 0 | 1 |  | 0 | 1 |
| *Proteus mirabilis* | 0 | 0 | 0 | 1 | 1 |
| *Acinetobacter spp* | 0 | 0 | 1 | 0 | 0 |
| *Pandorea pulmonicola* | 0 | 0 | 1 | 0 | 0 |
|  |  |  |  |  |  |
| Other microorganism |  |  |  |  |  |
| *Candida spp.* | 5 | 3 | 8 | 2 | 6 |

*CNS, Coagulase-negative staphylococci*

^a^ Four samples are missing

^b^ Blood culture were performed on central venous catheter, arterial line and post membrane oxygenator at each ECMO removal.
