# Supplementary material for: Venovenous extracorporeal membrane oxygenation devices-related colonisations and infections
Source: Ann Intensive Care. 2017 Nov 7;7:111. doi: 10.1186/s13613-017-0335-9 (PMC5676570; doi:10.1186/s13613-017-0335-9)
Supplement: Supplementary file 2 — Additional file 2. Table S2. Microorganisms cultured in blood culture (at the time of ECMO removal). [file 13613_2017_335_MOESM2_ESM.docx]

**Table S2:** Microorganisms cultured in blood culture

|  | Central venous catheter BC  n = 103 | Arterial catheter BC  n = 103 | Post membrane oxygenator BC  n = 103 |
| --- | --- | --- | --- |
| Gram positive |  |  |  |
| *CNS* | 4 | 6 | 1 |
| *Enterococcus spp* | 3 | 2 | 3 |
| *S. aureus* | 1 | 1 | 2 |
| *Propionobacterium acnes* | 0 | 0 | 1 |
| *Corynebacterium jeikeium* | 0 | 0 | 1 |
| Gram negative |  |  |  |
| *Enterobacter spp.* | 1 | 1 | 2 |
| *Pseudomonas aeruginosa* | 1 | 3 | 1 |
| *Klebsiella spp* | 1 | 1 | 0 |
| *Acinetobacter spp* | 1 | 0 | 0 |
| *Pandorea pulmonicola* | 0 | 0 | 1 |
|  |  |  |  |
| Other microorganism |  |  |  |
| *Candida spp.* | 2 | 3 | 3 |

BC, Blood culture: *CNS, Coagulase-negative staphylococci*
