# Supplementary material for: Venovenous extracorporeal membrane oxygenation devices-related colonisations and infections
Source: Ann Intensive Care. 2017 Nov 7;7:111. doi: 10.1186/s13613-017-0335-9 (PMC5676570; doi:10.1186/s13613-017-0335-9)
Supplement: Supplementary file 3 — Additional file 3. Table S3. Nosocomial infections during ECMO support of Extracorporeal Membrane Oxygenation (ECMO) in patients without infected/colonized ECMO device (U-I/C ED), with EMCO device colonization (ED-C) and ECMO device infection (ED-I) (at the time of ECMO removal). [file 13613_2017_335_MOESM3_ESM.docx]

**Table S3**: Multivariate analysis of factors associated with ECMO device infection (ED-I) or colonization (ED-C) at the time of ECMO removal.

|  |  | **U-I/C ED** | **ED-I + ED-C** | ***p* value^b^** | ***Hazard ratio (95%IC)*** | ***p value^c^*** |
| --- | --- | --- | --- | --- | --- | --- |
| **Number of ECMO^a^** |  | **60** | **43** |  |  |  |
| Sex Male |  | 35 (58.3) | 31 (72.1) | 0.151 | 2.275 (0.875-5.918) | 0.092 |
| BMI (kg/m^2^) |  | 24 (22-29) | 26 (24-31) | 0.074 | 1.056 (0.987-1.130) | 0.114 |
| Statin therapy |  | 8 (13.3) | 2 (4.7) | 0.187 | 2.827 (0.450-17.769) | 0.268 |
| Primary graft dysfunction (n, %) |  | 14 (23.3) | 3 (7.0) | 0.033 | 0.659 (0.072-6.018) | 0.712 |
| Pre-ECMO antibiotics^d^  (n, %) |  | 48 (80.0) | 39 (90.7) | 0.174 | 1.872 (0.302-11.605) | 0.501 |
| ECMO cannulation in ICU (n, %) |  | 44 (73.3) | 41 (98.3) | 0.004 | 7.815 (0.825-74.007) | 0.073 |
| Femoro-femoral cannulation (n, %) |  | 19 (31.7) | 5 (11.6) | 0.019 | 1.102 (0.228-5.318) | 0.904 |
| Per ECMO Plasma transfusion |  | 2 (0-8) | 0 (0-4) | 0.051 | 0.954 (0.888-1.024) | 0.192 |
| ECMO duration (days) |  | 7.5 (5-16) | 12 (9-18) | 0.005 | 1.021 (0.985-1.057) | 0.253 |

Table S4: Data are provided as No. (%) of ECMO or median value (interquartile range).

Abbreviations: BMI, Body mass index; ECMO, extracorporeal membrane oxygenation; ICU, Intensive care unit; IC, interval confidence.

U-I/C ED: Uninfected/Uncolonized ECMO device, ED-C: EMCO device colonization, ED-I: ECMO device infection

^a^  among the 99 patients, 4 underwent 2 ECMO during their ICU stay corresponding to 103 VV-ECMO

^b^ *p* value correspond to the univariate comparison between the Uninfected/Uncolonized ECMO device group (U-I/C ED) and the infected or colonized ECMO device group (ED-I + ED-C).

^c^ *p* value correspond to p value obtained by he the multinomial logistic regression procedure.

^d^pre-ECMO antibiotics correspond to antibiotics received for at least 24 hours before ECMO implantation
