# Supplementary material for: Venovenous extracorporeal membrane oxygenation devices-related colonisations and infections
Source: Ann Intensive Care. 2017 Nov 7;7:111. doi: 10.1186/s13613-017-0335-9 (PMC5676570; doi:10.1186/s13613-017-0335-9)
Supplement: Supplementary file 4 — Additional file 4. Table S4. Multivariate analysis of factors associated with ECMO device infection (ED-I) or colonization (ED-C) at the time of ECMO removal. [file 13613_2017_335_MOESM4_ESM.docx]

**Table S4**: Nosocomial infections during ECMO support of Extracorporeal Membrane Oxygenation (ECMO) in patients without infected/colonized ECMO device (U-I/C ED), with EMCO device colonization (ED-C) and ECMO device infection (ED-I).

|  | **Total ^a^** |  | **U-I/C ED** | **ED-C** | **ED-I** |
| --- | --- | --- | --- | --- | --- |
| **Number of ECMO** | **103** |  | **60** | **33** | **10** |
|  |  |  |  |  |  |
| Nosocomial infections^b^ | 44 |  | 19 | 22 | 3 |
| VAP | 20 |  | 8 | 11 | 1 |
| P-BSI/fungemia | 16 |  | 7 | 8 | 1 |
| Urinary tract infection | 6 |  | 3 | 3 | 0 |
| Surgical site infection | 1 |  | 0 | 0 | 1 |
| Acute cholecystitis | 1 |  | 1 | 0 | 0 |

Data are provided as No of ECMO

Abbreviations: ECMO, extracorporeal membrane oxygenation; P-BSI, Primary Bloodstream infection; VAP, Ventilator-Associated pneumonia

^a^ among the 99 patients, 4 underwent 2 ECMO during their ICU stay corresponding to 103 VV-ECMO

**^b^** Nosocomial infections excluding ECMO device infection
